# Counts of bovine monocyte subsets prior to calving are predictive for postpartum occurrence of mastitis and metritis

**DOI:** 10.1186/s13567-017-0415-8

**Published:** 2017-02-21

**Authors:** Brianna Pomeroy, Anja Sipka, Jamal Hussen, Melanie Eger, Ynte Schukken, Hans-Joachim Schuberth

**Affiliations:** 1 S3 119, Schurman Hall, College of Veterinary Medicine, Cornell University, Ithaca, NY 14850 USA; 2 Department of Population Medicine & Diagnostic Sciences, College of Veterinary Medicine, Cornell University, Ithaca, NY 14853 USA; 3 Immunology Unit, University of Veterinary Medicine Hannover, Foundation, 30173 Hannover, Germany; 4 Department of Microbiology and Parasitology, College of Veterinary Medicine and Animal Resources, King Faisal University, Al-Ahsa, Saudi Arabia; 5 Department of Physiology, University of Veterinary Medicine Hannover, Foundation, 30173 Hannover, Germany; 6 GD Animal Health, Deventer, The Netherlands; 7 Department of Animal Sciences, Wageningen University, Wageningen, The Netherlands

## Abstract

The heightened susceptibility to infectious diseases in postpartum dairy cows is often attributed to immune dysfunction associated with the transition period. However, the cell populations involved in this immune dysfunction and the dynamics between those populations are not well defined. Monocytes play a crucial role in governing initial immune response in bacterial infections. Bovine monocytes are subdivided in classical (CD14^+^/CD16^−^), intermediate (CD14^+^/CD16^+^) and non-classical monocytes (CD14^−^/CD16^+^) with distinct phenotypic and functional differences. This study investigated the relationship of monocyte subsets counts in blood at 42 and 14 days prior to expected calving date to occurrence of metritis and mastitis within 2 weeks postpartum. In the enrolled prospective cohort of 27 German Holstein cows, housed at the Institute of Animal Nutrition of the Friedrich-Loeffler-Institute Braunschweig, Germany, *n* = 13 developed metritis and/or mastitis postpartum. A multivariable logistic regression was used to analyze the relationship between prepartum cell counts of monocyte subsets and neutrophils with postpartum disease. Our model revealed that higher counts of the two CD14^+^ monocyte subsets were predictive of disease. In contrast, higher numbers of the CD14^−^ monocyte subset were negatively associated with disease. Interestingly, the neutrophil count, a common hallmark for inflammatory response, was not associated with the outcome variable at either time point. The results indicate that the number and composition of monocyte subsets before calving are related to the susceptibility to infectious disease within 2 weeks postpartum. Furthermore the oppositional effect of CD14^+^ and CD14^−^ subsets strengthens the hypothesis that these subsets have different functional roles in the inflammatory response in dairy cows.

**Electronic supplementary material:**

The online version of this article (doi:10.1186/s13567-017-0415-8) contains supplementary material, which is available to authorized users.

## Introduction

Dairy cows have an increased susceptibility to disease during the first 3 weeks postpartum [1]. Clinical mastitis and metritis are frequently observed in this period, and both of these diseases remain an animal welfare concern and a source of major costs for the dairy industry worldwide [2, 3]. The higher incidence of these diseases is commonly associated with an dysregulated inflammatory response in the animal, which often goes along with an overexpression of pro-inflammatory mediators like TNF-α and IL-1β and increased disease severity [4]. The innate immune system represents the first line of defense against the initial stages of infection and is the key determinant in the outcome of mastitis and metritis [5, 6]. Monocytes and monocyte-derived cells play an essential role in orchestrating the main features of the innate immune response [7, 8]. Recently it has become evident that bovine monocytes are a heterogeneous population and can be divided based on the expression of CD14 and CD16 into classical (cM, CD14^+^/CD16^−^), intermediate (intM, CD14^+^/CD16^+^) and non-classical monocytes (ncM, CD14^−^/CD16^+^), with the majority of blood monocytes being CD14 positive [9]. Distinct functional features were reported for the different subsets in response to LPS and in the presence of chemokines. Hussen et al. [9] found short term LPS stimulation (3 h) induced stronger pro-inflammatory cytokine response and inflammasome activation in CD14^+^ subsets (cM and intM) compared to the CD14^−^ ncM; however, Corripio-Miyar et al. [10] found that ncM showed an overall stronger pro-inflammatory response after long term LPS (18 h) stimulation. In conjunction with inflammatory responses to LPS, the cM also are able to produce relatively strong anti-inflammatory responses including high arginase I gene expression and IL-10 production. Only the CD14^+^ subsets, cM and intM, have been reported to be attracted by both the monocyte chemokine CCL5 and neutrophil degranulation products in vitro and to respond with significant Ca^2+^ influx [11, 12]. Though there remains some discrepancies in function in regards to CD14^−^ monocytes it is evident these subsets have unique properties and CD14^+^ consistently show strong inflammatory responses.

Although the role and dynamics of the different bovine monocyte subsets in vivo remain unclear, it has been shown that cows that develop infectious disease postpartum also have an altered monocyte response. Periparturient cows showed an overall higher frequency of monocytes in supra mammary lymphnodes and a higher TNF-α production in tissue monocytes after stimulation with LPS but significantly lower response in blood monocytes [13]. Monocytes from cows which developed metritis within the first week postpartum showed elevated baseline expression of pro-inflammatory cytokines such as IL-6, TNF-α and IL-1β at 1 to 12 h post calving, but showed a less pronounced increase of these mediators in response to bacterial challenge in vitro [14]. Despite an enhanced baseline pro-inflammatory cytokine production those cows could not prevent the establishment of infection with rapid clearance of the invading pathogen, and increased inflammatory properties of these cells likely promoted disease. Another study showed that monocyte phagocytic capacity was significantly decreased in cows that developed endometritis within the first 20 days postpartum [15]. It is unclear from these studies if susceptible cows also show changes in monocyte subset composition or an altered function of individual monocyte subsets. In humans however, it has been shown that there are associations between inflammatory conditions and monocyte subset composition. In tuberculosis patients an expansion of CD16^+^ subsets was reported while in vitro CD16^−^ monocyte subsets were more prone to migrate in response to mycobacteria derived gradients [16]. In an endotoxin tolerance model with human monocytes it was furthermore shown that prior exposure to LPS increased the frequency of CD14^+^ monocyte subsets after LPS restimulation [17]. Additional studies showed that human CD14^−^ ncM were the most inflammatory of the subsets, and an increase in ncM was associated with autoimmune disorders and promotion of Th1-type responses, whereas CD14^+^ cM and intM have been found to attenuate T cell responses and were associated with certain diseases in humans such as sepsis and certain cancers [18, 19]. Research pertaining to relationships between disease and monocyte subsets is underdeveloped in bovine immunology, yet the substantial evidence connecting disease and monocyte composition found in other species in conjunction with known species to species differences indicates this area should be further investigated within a bovine model.

Therefore, the objective of this study was to investigate if there were a relationship between prepartum blood monocyte subset composition and postpartum infectious disease in dairy cows. Specifically, we investigated if disease susceptibility was correlated with changes in either CD14^+^ or CD14^−^ subsets. We used logistic regression models with longitudinal prospective data to identify significant relationships between postpartum disease and counts of monocyte subsets.

## Materials and methods

### Animals and blood sampling

The data was collected from cows enrolled in a previously published study [20]. Briefly, 27 German Holstein cows housed at the experimental station of the Institute of Animal Nutrition, Friedrich-Loeffler-Institute in Braunschweig, Germany were enrolled in the study. Body condition score (BCS) was determined prior to the experiment and cows were allotted to a BCS low (BCS 2.77 ± 0.10, mean ± SEM) and a BCS high group (BCS 3.73 ± 0.12) with consideration of milk yield, body weight and lactation number (Table 1). Throughout the experiment the animals were housed in a free-stall barn with individual feeding stations. After calving cows were milked twice per day. Pre-partum, BCS low cows received a diet of 80% roughage, 50% grass silage and 50% corn silage based on dry matter content, and 20% concentrate according to the recommendations of the German Society of Nutrition Physiology (GfE, 2001). The BCS high group received 40% of the same roughage and 60% concentrate to induce energy oversupply. After calving the concentrate proportion in the diet was raised from 30 to 50% in 2 or 3 weeks for the BCS low and the BCS high group, respectively, to promote negative energy balance in the BCS high group. Half of the enrolled animals were vaccinated against BVD (Bovilis BVD^®^-MD, MSD) at 42 and 14 days prior to expected calving date; half of the low BCS was vaccination and approximately half of the high BCS was vaccinated. The number of cows with a parity of 2 and cows with a parity of greater than 2 were approximately the same between the groups i.e. four groups based on BCS and vaccination status. Health status was monitored throughout the trial. Cows with purulent uterine discharge detected in the vagina were diagnosed with clinical metritis based on the definition provided by Sheldon et al. [21]. Mastitis cases were identified by clinical signs, i.e. clots in milk, elevated somatic cell count, swelling/sensitivity of the mammary gland. Bacteriological cultures were not preformed to determine the etiology of clinical disease; metritis and mastitis were defined by clinical signs. For the purpose of this paper animals that developed metritis and/or mastitis within two weeks postpartum were combined into a single category of postpartum disease; therefore a binary outcome variable of postpartum disease yes or no was used in the subsequent data analyses (Table 1). Three cows developed clinical mastitis only, seven cows developed metritis only, and three cows developed both mastitis and metritis in the first 2 weeks postpartum. Blood samples were collected by puncture of the jugular vein into heparinized vacutainer tubes at days 42 and 14 prior to predicted calving date. Only two cows calved more than 2 weeks prior to predicted parturition, their prepartal sample was assigned to the nearest predicted day (one cow day−42, one cow day−14). This study was approved by the Lower Saxony State Office for Customer Protection and Food Safety (33.9–42502–04–11/0444). All procedures involving animals were carried out in accordance with the German legislation on animal welfare.

**Table 1:** **Descriptive analysis and characteristics of enrolled population**

Parameter	Healthy (*n* = 14)	Postpartum disease (*n* = 13)
	Frequency	Frequency
Parity = 2	5	7
Parity > 2	9	6
BCS < 3	7	6
BCS > 3	7	7
Unvaccinated	8	8
Vaccinated	6	5

	Median	IQR	Median	IQR
42 days prior to calving (cells/µL)				
Neutrophil absolute count	3075.5	2149	2851	1790
CD172a^+^ absolute cell count	517.90	171.16	552.15	616.21
CD14^+^ monocyte absolute count	469.95	170.13	510.12	557.28
CD14^−^ monocyte absolute count	33.45	27.06	32.73	37.63
14 days prior to calving (cells/µL)				
Neutrophil absolute count	3884.75	2303	4326.05	2202
CD172a^+^ absolute cell count	389.86	680.35	1003.21	399.70
CD14^+^ monocyte absolute count	341.98	602.29	915.14	358.69
CD14^−^ monocyte absolute count	36.21	60.53	53.46	49.16

Median cell counts with interquartile range (IQR) presented.

### Separation of blood leukocytes and characterization of monocyte subsets

Blood was diluted with the same volume of PBS and centrifuged at 1000 × *g* for 10 min. Erythrocytes were lysed by adding 20 mL aqua dest. for 20 s and subsequent addition of 20 mL double concentrated PBS. This was repeated twice until complete erythrolysis. Cells were centrifuged and washed with PBS (500 × *g*, 250 × *g* and 100 × *g* for 10 min each) and finally adjusted to 1 × 10^7^ cells/mL in PBS. Leukocytes were suspended in PBS containing 5 g/L bovine serum albumin and 0.1 g/L NaN_3_ (MIF buffer) and stained with a combination of three directly conjugated monoclonal antibodies: mouse anti-bovine CD172a-PECy5, mouse anti-human CD14-PE and mouse anti-human CD16-FITC (all from AbD Serotec, Oxford, UK) for 20 min at 4 °C. Thereafter cells were washed with MIF buffer and analyzed by flow cytometry (Accuri C6 Flow Cytometer^®^, Becton–Dickinson GmbH, Heidelberg, Germany). Dead cells were excluded by adding propidium iodide (2 μg/mL, Calbiochem, Bad Soden, Germany). Mononuclear cells (MNC) and granulocytes (PMN) were gated according to their forward (FSC) and side scatter (SSC) properties [22]. Among CD172a^+^ MNC, three bovine monocyte subsets were defined based on their CD14 and CD16 expression: cM were CD14^+^/CD16^−^, intM were CD14^+^/CD16^+^ and ncM CD14^−^/CD16^+^. Appropriate compensation was applied for fluorochromes used in multi-color flow analysis of monocyte subsets in order to distinguish between PI and PE. Cell doublets were gated out in dot plots SSC-A vs SSC-H. Cell counts of monocyte subsets and PMN were calculated by multiplying the absolute leukocyte count, determined in EDTA whole blood using an automatic analyzer (Celltac α MEK-6450, Nihon Kohden, Qinlab Diagnostik, Weichs, Germany), with percentages determined by flow cytometry.

### Data analysis and statistical methods

All data were entered into a database and double checked for entry errors or outliers. Data were described using descriptive and graphical techniques. Descriptive analysis of raw data included the computation of median cell counts with interquartile range for individual cell populations measured at each sample time point and frequency tables of categorical study design variables (vaccination status, BCS, parity) and grouped by postpartum disease status. The small sample size precluded univariable statistical analyses of any associations between disease presence and BCS, parity, or vaccination status. Spearman’s correlation coefficients were calculated to identify correlations between counts of different myeloid cell populations to assess for possible collinearity. Further analysis was performed using multivariable regression analyses. The general logistic regression model was formulated as: Logit(Y) = α + β_i_ X_i_ + e, where Y is the absence or presence of postpartum disease, α is the intercept, β_i_ is the regression coefficient of predictor variable X_i_. The term e is an independently, identically distributed binomial error term. Statistical significance was defined at *P* < 0.05. All Statistical analyses were preformed using SAS version 9.4 (SAS Institute). The dataset supporting the conclusions of this article is included within the article as an additional file (see Additional file 1).

### Multivariable analysis

Multiple multivariable logistic models were used to assess the relationship between absolute counts of different myeloid cell populations circulating in the peripheral blood during the third trimester of pregnancy (42 days and 14 days prior to calving) and development of disease within the first two weeks postpartum. Multivariable logistic models were constructed using generalized estimating equations and a binary distribution for the outcome variable (postpartum disease, yes vs. no). Potential confounding variables included in the model were the original study design variables parity (parity = 2, parity >2), body condition score (>3.0, <3.0), and vaccination schedule (vaccinated prepartum, yes = 1 vs. no = 0). These potential confounders were included in all multivariable logistic models as binary variables. Due to differences observed in counts of multiple myeloid cell populations between 42 and 14 days prior to calving date, two logistic models were constructed based on time of data collection as a minimal model irrespective of individual contributions. The two models included data from either 42 days or 14 days prior to calving; any animals with missing data from either time point were excluded. Final models were generated using a forward stepwise selection of variables. Statistical significance was determined based on the likelihood ratio statistic of nested models, and model fit was described using Akaike’s Information Criterium (AIC). Likelihood ratios and odds ratio estimates with profile-likelihood confidence intervals were used to determine significance due the small sample size. With the data analyzed here, a Wald test is not preferable over the likelihood ratio test because the estimates for the coefficient and its standard error may have unreliable normal approximation of its distribution when the sample size is small; likelihood ratio test and profile-likelihood confidence intervals do not assume normality of the estimator [23].

## Results

### Descriptive analysis

**Figure 1:**
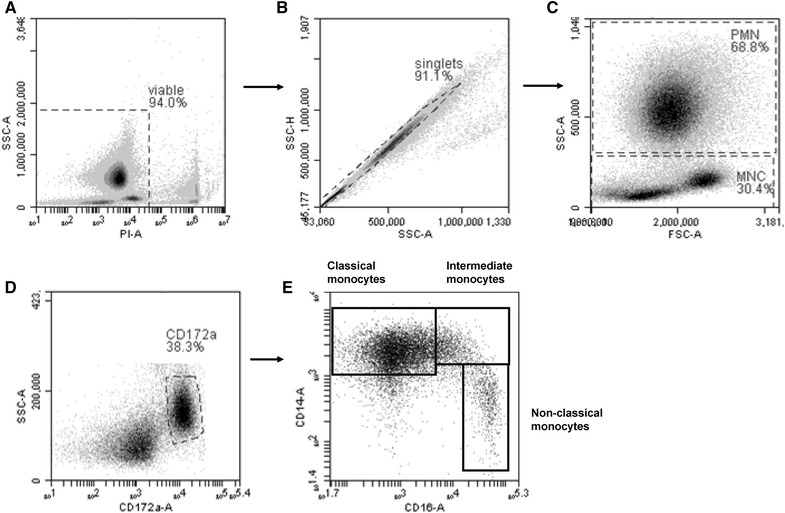
**Gating strategy utilized for flow cytometric analysis of bovine peripheral blood leukocytes showing representative data from one animal.** After gating on viable (propidium-iodide-negative) cells (**A**), cell doublets were excluded by SSC-A and SSC-H gating (**B**). Bovine mononuclear cells (MNC) and granulocytes (PMN) were gated based on their forward and side scatter characteristics and their percentages were calculated (**C**). Three-color immunofluorescence of bovine MNC with mAbs to CD172a, CD14 and CD16 defines three monocyte subsets in peripheral blood. Viable mononuclear cells, based on forward and side scatter characteristics, were gated on CD172a-positive cells (**D**). Dot plots of CD14 and CD16 expression display classical monocytes (CD14^+^CD16^−^, upper left), intermediate monocytes (CD14^+^CD16^+^, upper right) and nonclassical monocytes (CD14^−^CD16^+^, lower right) (**E**)

**Figure 2:**
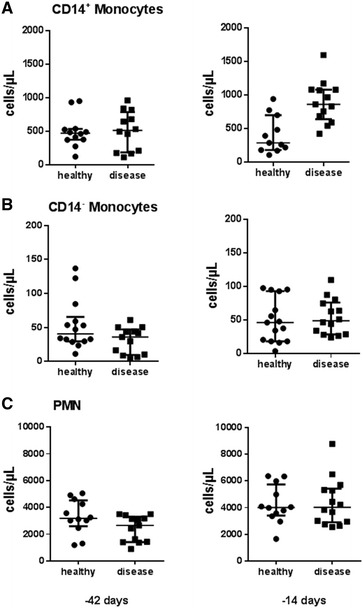
**Monocyte and neutrophil counts in blood prepartum.** Absolute counts of CD14^+^ monocytes (**A**) and CD14^−^ monocytes (**B**) and PMN (**C**) per µL blood at 42 and 14 days prior to expected calving date in 27 multiparous cows. 13 cows developed mastitis and/or metritis within 14 days postpartum (square symbols), 14 cows did not develop either disease (circular symbols). Individual values are shown with median and interquartile range.

Characteristics of the enrolled cows are summarized in Table 1. The animals were grouped by parity, BCS and vaccine status, and the model controlled for these host characteristics though addition of forced variables. Out of the 27 enrolled cows 13 developed clinical disease postpartum. There were comparable numbers of cows within each category of BCS (either > or <3), parity (= 2, >2), and vaccinated between cows who did not develop clinical disease postpartum (“healthy”) and those cows who did develop clinical disease postpartum (“postpartum disease”) as determined by the Chi square test (Table 1). The gating strategy used to determine absolute count of CD14^−^ monocytes [CD172a^+^CD14^−^ mononuclear cells (MNC)], CD14^+^ monocytes (CD172a^+^CD14^+^ MNC), and granulocytes (PMN) in blood is presented in Figure 1. The counts of CD14^+^ monocytes, CD14^−^ monocytes, and PMN in blood at 42 days and 14 days prior to the expected calving date within healthy cows and postpartum disease cows are presented in Table 1 and Figure 2. Cows that developed postpartum disease had a median CD14^+^ subset count at 14 days prior to expected calving date of 863 cells/µL blood whereas counts of CD14^+^ subsets in cows that did not develop disease postpartum had median count of only 289 cells/µL blood (Figure 2A). The healthy and postpartum disease group had comparable median counts of CD14^−^ subsets of the for both time points (Figure 2B). Descriptive analysis of monocyte subset counts based on parity revealed that cows in parity 2 that developed postpartum disease had median CD14^+^ and CD14^−^ counts of 677 and 44 cells/μL, respectively whereas cows in parity 3 or greater had median CD14^+^ and CD14^−^ counts of 174 and 9 cells/μL, respectively, validating the need to utilize a model to elucidate relationships between disease and monocyte populations so that these cofounders can be controlled for Additional file 2. The counts of PMN showed similar dynamics in healthy and postpartum disease cows in blood at 42 and 14 days prior to expected calving date (Table 1; Figure 2C).

Based on collinearity of cM and intM identified by Spearman’s correlation coefficients and similar biological function (Hussen et al. [9]) between these two CD14^+^ subsets, we chose to group CD14^+^ subsets into a single variable of absolute count of all CD14^+^ monocytes. Only ncM are CD14^−^, and thus absolute count of CD14^−^ monocytes included only ncM.

### Multivariable regression models

**Figure 3:**
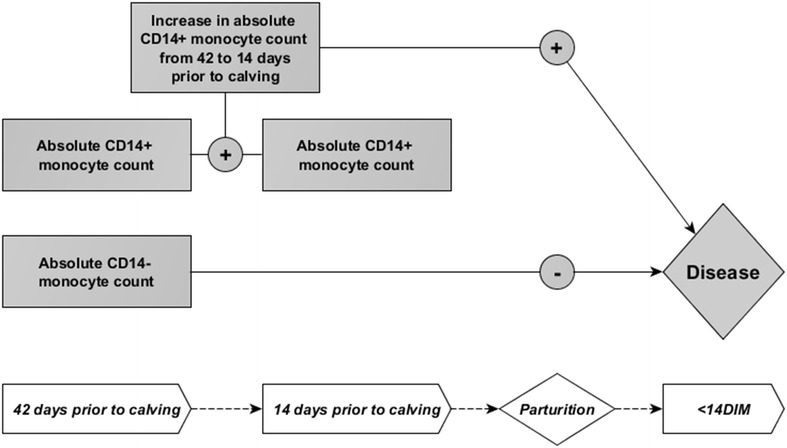
**Results from final model selected for data from 42** **days prior to calving date.** Greater increase in peripheral CD14^+^ monocyte counts from 42 days prior to 14 days prior to expect calving date associated with increased risk of postpartum disease <14 days post-calving (increased risk indicated as “+”). Higher peripheral CD14^−^ monocyte counts at 42 days prior to expected calving date associated with a decreased risk of postpartum disease <14 days post-calving (decreased risk indicated as “−”).

**Figure 4:**
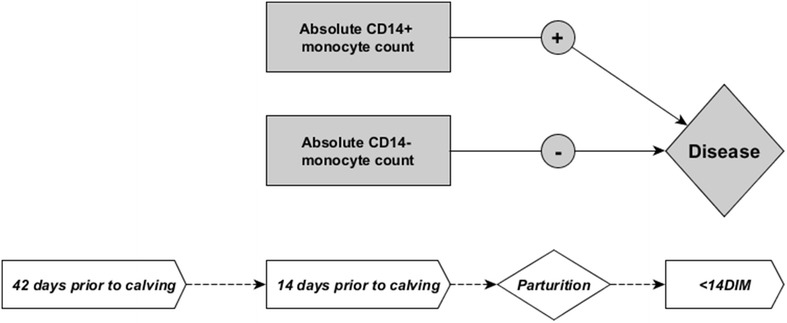
**Results from final model selected for data from 14** **days prior to calving.** Higher peripheral CD14^+^ monocyte counts at 14 days prior to expect calving date associated with increased risk of postpartum disease <14 days post-calving (increased risk indicated as “+”). Higher peripheral CD14^−^ monocyte counts at 14 days prior to expected calving date associated with a decreased risk of postpartum disease <14 days post-calving (decreased risk indicated as “−”).

Based on these descriptive analyses two separate models were generated for data from 42 days prior to expected calving date and 14 days prior to expected calving date and changes in cell counts over the days were also analyzed. At 42 days prior to the expected calving date, the final model selected with a forward stepwise selection included the change in absolute counts of CD14^+^ monocytes from 42 days to 14 days prior to expected calving date (Δ_14d-42d_ CD14^+^), and absolute count of CD14^−^ monocytes at 42 days prior to expected calving date (Tables 2, 3, 4; Figure 3). At 14 days prior to the expected calving date, the final model selected with a forward stepwise selection included the absolute count of CD14^+^ monocytes and absolute count of CD14^−^ monocytes at 14 days prior to expected calving date (Tables 5, 6, 7; Figure 4). A flow chart summarizing the models findings on the statistically significant relationships between 42 days and 14 days prepartum absolute cell counts and postpartum disease are depicted in Figures 3 and 4, respectively; parameters which decreased the risk of acquiring a postpartum disease are indicated with “−” and those which increased the risk of acquiring postpartum disease are indicated with “+”.

**Table 2:** **Multivariable analysis, Model 1 selection: only data collected at 42** **days prior to calving date used to generate model**

42 days prior to calving model	−2Loglikelihood	AIC	Likelihood ratio	*P* value
Base model: parity+ BCS+ vaccination status	28.89	36.89	–	–
Model 1a: parity + BCS + vaccination status + ΔCD14^+^	20.63	30.63	8.27	0.004
Model 1b: parity + BCS + vaccination status + ΔCD14 ^++^ CD14^−^	11.80	23.80	8.83	0.003
Model 1c: parity + BCS + vaccination status + CD14^+^/CD14^−^ ratio	22.54	32.54	6.36	0.012

Model 1b is the best fitting model.

**Table 3:** **Parameter estimates for final model selected for 42** **days prior to expected calving date**

Parameter	β	Standard error	χ^2^	*P* value
Intercept	−6.2125	2.953	–	–
Parity (>2)	5.9317	2.8295	8.91	0.0028
Parity (=2, reference)	0	0	–	–
BCS (low, <3)	−0.4746	1.9196	0.06	0.8
BCS (high, >3, reference)	0	0	–	–
Vaccine (yes)	0.6141	1.5264	0.16	0.6879
Vaccine (no, reference)	0	0	–	–
CD14^−^ monocyte count (cells/µL)	−0.1135	0.0615	8.83	0.003
Δ_14d–42d_ CD14^+^	0.0059	0.0032	7.75	0.0054

Exact *p*-values calculated using a χ^2^ distribution; significance based on the likelihood ratio test.

**Table 4:** **Odds ratio estimates and profile-likelihood confidence intervals for significant explanatory variables from final model selected for 42** **days prior to expected calving date**

Parameter	Unit	Odds ratio estimate	95% confidence limits	
CD14^−^ monocyte count (cells/µL)	5.0000	0.567	0.213	0.860
Δ_14d–42d_ CD14^+^	200.0	3.270	1.304	18.950

Unit refers to the change in number of units the odds ratio estimate was based.

**Table 5:** **Multivariable analysis, Model 2: Only data collected at 14** **days prior to calving date used to generate model**

14 days prior to calving model	−2Loglikelihood	AIC	Likelihood ratio	*P* value
Base model: parity + BCS + vaccination status	28.89	36.89	–	–
Model 2a: parity + BCS + vaccination status + CD14^+^	17.68	27.68	11.21	0.0008
Model 2b: parity + BCS + vaccination status + CD14^++^ CD14^−^	8.45	20.45	9.23	0.0024
Model 2c: parity + BCS + vaccination status + CD14^+^/CD14^−^ ratio	20.82	30.82	8.087	0.0045

Model 2b was the best fitting model.

**Table 6:** **Parameter estimates for final model selected for 14** **days prior to expected calving date**

Parameter	β	Standard error	χ^2^	*P* value
Intercept	−7.7997	5.2934	–	–
Parity (= 2)	1.5204	2.1404	0.61	0.4346
Parity (> 2, reference)	0	0	–	–
BCS (low, < 3)	−11.753	7.5015	8.86	0.0029
BCS (high, > 3, reference)	0	0	–	–
Vaccine (yes)	−9.9861	6.845	5.67	0.0173
Vaccine (no, reference)	0	0	–	–
CD14^−^ monocyte count (cells/µL)	−0.2536	0.1658	9.23	0.0024
CD14^+^ monocyte count (cells/µL)	0.044	0.0272	20.44	<.0001

Exact *p*-values calculated from likelihood ratio using a χ^2^ distribution; significance based on the likelihood ratio test.

**Table 7:** **Odds ratio estimates and profile-likelihood confidence intervals for significant explanatory variables from final model selected for 14** **days prior to expected calving date**

Parameter (cells/µL)	Unit	Odds ratio estimate	95% confidence limits	
CD14^−^ monocyte count	5.0000	0.281	0.016	0.811
CD14^+^ monocyte count	50.0000	9.033	1.591	635.660

Unit refers to the change in number of units the odds ratio estimate was based.

The absolute count of CD14^−^ monocytes at 42 days and 14 days prior to expected calving date were significantly associated with a reduced risk of acquiring postpartum disease (Tables 2, 3, 4, 5, 6 7; Figures 3, 4). The odds ratio estimate for CD14^−^ monocyte count was calculated based on if the count in blood were to increase by 5 cell/μL due to the relatively low numbers typically found in circulation; these odds ratio estimates were 0.567 and 0.281 at 42 days and 14 days prior to expected calving dates, respectively (Tables 4, 7). Models showed the increase in Δ_14d–42d_ CD14^+^ monocyte count and the absolute counts of CD14^+^ monocytes at 14 days prior to expected calving date were significantly associated with increased risk of postpartum disease (Tables 2, 3, 4, 5). The odds ratio estimate for Δ_14d–42d_ CD14^+^ monocyte count was calculated based on if the Δ_14d–42d_ CD14^+^ monocyte count were to increase by 200 cell/μL due to the relatively high numbers of CD14^+^ monocytes typically found in circulation and changes observed between 42 and 14 days prior to the expected calving date; the odds ratio estimate was 3.270 for Δ_14d–42d_ CD14^+^ monocyte count at 42 days prior to expected calving date (Table 4). The odds ratio estimate for CD14^+^ monocyte count was calculated based on if the count in blood were to increase by 50 cell/μL due to the relatively high numbers typically found in circulation; the odds ratio estimate was 9.033 for CD14^+^ monocyte count at 14 days prior to expected calving date (Table 7). Interestingly, absolute count of PMN circulating in the blood was not found to be a significant predictor of postpartum disease (metritis and mastitis) in either model selected using data from 42 days or 14 days prior to expected calving date given the data on monocyte subsets (Tables 2, 5). The original study design variables for parity, BCS, and vaccination status included as confounding variables in the model were found to have significant relationships with disease, although these relationships were not significant at all time points; a parity of greater than 2 increased disease risk in the model for −42 days samples, a BCS less than 3 decreased risk of disease in the model for −14 days samples, and vaccination in the prepartum period decreased risk of disease in the model for −14 days samples (Tables 3, 6).

## Discussion

Based on the multivariable models described here, the composition of monocyte subsets in peripheral blood differs in cows that develop disease within 2 weeks postpartum compared to cows that remain healthy. Changes in the numbers of CD14^−^ monocytes and CD14^+^ monocytes in the periphery predict the development of postpartum disease (mastitis, metritis). Higher counts of circulating CD14^−^ monocytes at 42 and 14 days prior to calving reduced the probability of acquiring postpartum disease, whereas, an increase in CD14^+^ monocyte counts from 42 to 14 days and the CD14^+^ monocyte counts at 14 days prior to calving increased the probability of acquiring postpartum disease (Tables 3, 5; Figures 2, 3). The results of the regression analyses were presented in a pictorial way in Figures 2 and 3. A “+” in the figures represents a higher risk of disease whereas a “−” represents a lower risk. These positive or negative indicators of disease risk correspond to positive or negative value of the regression coefficient, β, in Tables 3 and 5. The CD14^+^ subsets account for the majority of monocytes found in circulation with cM being the dominating cell population predicting an increased disease risk. As mentioned previously, the two bovine CD14^+^ subsets (cM, intM) have overlapping functions that differ from the CD14^−^ monocyte subset (ncM). For example, both cM and intM respond to neutrophil degranulation products, migrate in presence of CCL5, and have high phagocytic capacity, whereas ncM do not [9, 11, 12]. Nonclassical monocytes have been described as being the most mature of all monocytes subsets [9]. Bovine ncM have been shown to have limited capacity to respond to LPS with inflammation or neutrophil degranulation products compared to the other subsets [9, 11, 12]. In humans, ncM have a unique ability to patrol healthy tissue, and bovine ncM have recently been shown to express adhesion molecules associated with similar behavior, suggesting that ncM are the first of the monocyte subsets to recognize and respond to infection [12, 24]. Therefore changes in peripheral monocyte composition could lead to disturbances in primary recognition of invading pathogens within tissue as well as in the balance of specific inflammatory/anti-inflammatory responses to follow. In non-bovine species, diet composition, circadian rhythm, glucocordicoids, and vitamin D3 have all been shown to modulate blood monocyte subset composition, but there are currently no published studies addressing the regulation of blood monocyte composition in cattle, thus it cannot be speculated at this time what factors may cause the differences we observed [25–27]. Nonetheless, prepartum blood monocyte composition appears to be correlated with postpartum immune competency.

Peripheral monocytes migrate into tissues either in steady state or during inflammation where they may differentiate into macrophages or dendritic cells. Similar to rodents and humans, in bovine pregnancy, macrophages accumulate in the endometrium and are present in large numbers in interplacentomal and placentomal endometrium; CD14^+^ cells in circulation share similar gene expression profiles to macrophages in the endometrium, indicating blood monocytes likely migrate to endometrium and differentiate into macrophages [28, 29]. Tissue-resident macrophages and recruited monocytes play an important role in tissue repair in the reproductive tract postpartum. Within late gestation and dry period (the non-lactating period of late gestation in cows) there is a significant increase in the number of macrophages in both the reproductive tract and mammary gland in dairy cattle [28–31]. The composition of tissue-resident monocytes and monocyte derived cells in the reproductive tract and mammary gland during bovine late gestation is likely dependent on the available reservoir, migratory capacity, and functional differences of the monocyte composition in blood therefore, changes in monocyte populations likely influence disease susceptibility [11, 12, 32, 33]. Future work should investigate the hypothesized relationship between blood monocyte composition and tissue resident monocyte and macrophage populations and their individual roles in shaping immune response to invading pathogens.

Though no concrete conclusions can be made on the exact role of these subsets in bovine postpartum disease at this time, this is the first piece of striking evidence which clearly shows a relationship between monocyte subset composition prepartum and postpartum disease and alludes to the different roles of CD14^+^ and CD14^−^ subsets in disease susceptibility.

In contrast to others who described relationships between neutrophil function in the periparturient period, negative energy balance and metritis postpartum, this model found no relationship between neutrophil counts in the peripheral blood at 42 and 14 days prior to calving and postpartum disease [34, 35]. The data used generate the models presented here do not include data on functional properties of either neutrophils or monocyte subsets from these animals. Though prepartum numbers of neutrophils readily available to migrate into tissue were not associated with postpartum disease in these animals like monocyte subsets are, the risk of disease may still be impacted by neutrophil function like described in previous studies [35–37]. The original study design variables for parity, BCS, and vaccination status had significant relationships with disease, although these relationships were not significant at all time points the relationships were biologically plausible based on peer-reviewed literature on trained immunity from vaccination, the role of metabolic factors in disease, and relationships between parity and immune function and disease; prepartum vaccination, lower BCS (<3), and animals of a parity of 2 had decreased risk of disease (Tables 3, 5) [38–40]. For example, Gilbert et al. [40] found cows with a parity 4 or greater had reduced neutrophil function in the periparturient period.

Our results clearly indicate that peripheral blood monocyte composition relates to the overall immune competence of the cow postpartum. Though exact mechanisms which heighten susceptibility to postpartum disease cannot not be determined by this model, immune dysfunction postpartum seems to become apparent by blood monocyte composition as early as 42 days prepartum. These findings provide new insight on maternal immune status and its relationship to postpartum disease. Prepartum monocyte composition could be a potential biomarker to identify cows at risk with compromised immune function, and therefore could aid in animal selection for immunomodulation therapies. Future research should address the specific roles of bovine monocyte subsets in disease and address findings from the model described here.

The model presented here demonstrates that changes in the numbers of CD14^−^ monocytes and CD14^+^ monocytes in the periphery prepartum predict the development of postpartum disease (mastitis, metritis). We found higher counts of circulating CD14^−^ monocytes at 42 and 14 days prior to calving reduced the probability of acquiring postpartum disease, whereas, an increase in CD14^+^ monocyte counts from 42 to 14 days/at 14 days prior to calving increased the probability of acquiring postpartum disease. Our findings highlight the need for further investigation on the function and regulation of individual monocyte subsets in pregnancy and early lactation in order to elucidate their role involved in disease susceptibility. A greater understanding of late gestation immune status and its relationship to postpartum disease will allow for development of immunomodulators and improvements in dry cow management that will reduce the risk of postpartum disease.

## Additional files

**Additional file 1.** **Complete dataset**. This file contains a table of the complete dataset from all enrolled cows used for descriptive analysis and statistical analysis (Materials and methods).

**Additional file 2.** **Cell counts by parity**. This file contains a table summarizing descriptive analysis of cell count by parity group (Results).
