# Supplementary material for: Counts of bovine monocyte subsets prior to calving are predictive for postpartum occurrence of mastitis and metritis
Source: Vet Res. 2017 Feb 21;48:13. doi: 10.1186/s13567-017-0415-8 (PMC5320682; doi:10.1186/s13567-017-0415-8)
Supplement: Supplementary file 1 — Additional file 1. Complete dataset. This file contains a table of the complete dataset from all enrolled cows used for descriptive analysis and statistical analysis (Materials and methods). [file 13567_2017_415_MOESM1_ESM.docx]

**Additional file 1** **Complete data set used for descriptive and statistical analysis.** Abbreviations: Disease (0 = no disease, 1 = disease recorded postpartum), individual cow identification number (Cow ID), time point (TP, −42 = 42 days prior to expected calving date, −14 = 14 days prior to expected calving date), absolute counts of classical monocytes (cM), absolute counts of intermediate monocytes (intM), absolute counts of non-classical monocytes (ncM), body condition score (BCS, 1 = BCS < 3, 2 = BCS > 3), Parity (1 = parity = 2, 2 = parity > 2), absolute counts of CD172+ CD14+ cells (CD14pos), absolute counts of CD172a+CD14- cells (CD14neg), absolute counts of PMN (Neutrophil), vaccine status (Vacc, 0 = no BVD prepartum vaccination, 1 = yes BVD prepartum vaccination).

| Disease | Cow ID | TP | cM | intM | ncM | BCS | Parity | CD14pos | CD14neg | Neutrophil | Vacc |
| --- | --- | --- | --- | --- | --- | --- | --- | --- | --- | --- | --- |
| 1 | 2 | -42 | 703 | 133 | 50 | 1 | 1 | 836 | 50 | 2435.3 | 0 |
| 1 | 2 | -14 | 854 | 114 | 63 | 1 | 1 | 968 | 63 | 4212.1 | 0 |
| 0 | 7 | -42 | 482 | 79 | 43 | 1 | 2 | 561 | 43 | 1306.5 | 1 |
| 0 | 7 | -14 | 741 | 167 | 51 | 1 | 2 | 908 | 51 | 2963.6 | 1 |
| 1 | 20 | -42 | 448 | 81 | 30 | 1 | 1 | 529 | 30 | 3050 | 0 |
| 1 | 20 | -14 | 788 | 74 | 28 | 1 | 1 | 863 | 28 | 5282 | 0 |
| 1 | 27 | -42 | 581 | 61 | 36 | 2 | 1 | 643 | 36 | 3135.6 | 0 |
| 1 | 27 | -14 | 445 | 242 | 81 | 2 | 1 | 688 | 81 | 6497 | 0 |
| 1 | 30 | -42 | 169 | 38 | 44 | 1 | 1 | 207 | 44 | 1400 | 1 |
| 1 | 30 | -14 | 996 | 84 | 64 | 1 | 1 | 1080 | 64 | 3670.4 | 1 |
| 0 | 32 | -42 | 759 | 171 | 53 | 2 | 1 | 929 | 53 | 4914 | 0 |
| 0 | 32 | -14 | 230 | 32 | 18 | 2 | 1 | 262 | 18 | 6336 | 0 |
| 0 | 35 | -42 | 490 | 45 | 47 | 2 | 1 | 535 | 47 | 4253.4 | 0 |
| 0 | 35 | -14 |  |  |  | 2 | 1 |  |  |  | 0 |
| 0 | 37 | -42 | 419 | 62 | 34 | 2 | 1 | 481 | 34 | 3019.4 | 1 |
| 0 | 37 | -14 | 189 | 33 | 38 | 2 | 1 | 222 | 38 | 3785.5 | 1 |
| 0 | 38 | -42 | 146 | 127 | 122 | 2 | 1 | 273 | 122 | 1189 | 1 |
| 0 | 38 | -14 | 890 | 142 | 95 | 1 | 1 | 1032 | 95 | 4043.2 | 1 |
| 1 | 46 | -42 | 823 | 135 | 41 | 1 | 1 | 958 | 41 | 3180.8 | 1 |
| 1 | 46 | -14 | 912 | 261 | 24 | 1 | 1 | 1173 | 24 | 2752 | 1 |
| 0 | 47 | -42 | 776 | 171 | 85 | 1 | 1 | 947 | 85 | 4617 | 0 |
| 0 | 47 | -14 | 803 | 140 | 96 | 1 | 1 | 943 | 96 | 3984 | 0 |
| 1 | 50 | -42 | 633 | 45 | 61 | 2 | 1 | 677 | 61 | 3492 | 0 |
| 1 | 50 | -14 | 897 | 174 | 88 | 2 | 1 | 1071 | 88 | 4440 | 0 |
| 1 | 618 | -42 | 98 | 11 | 9 | 2 | 2 | 109 | 9 | 3425 | 0 |
| 1 | 618 | -14 | 678 | 79 | 29 | 2 | 2 | 757 | 29 | 5328 | 0 |
| 0 | 716 | -42 | 344 | 65 | 33 | 1 | 2 | 409 | 33 | 3254.7 | 0 |
| 0 | 716 | -14 | 97 | 12 | 4 | 1 | 2 | 109 | 4 | 5987.8 | 0 |
| 0 | 736 | -42 |  |  |  | 2 | 2 |  |  |  | 1 |
| 0 | 736 | -14 | 669 | 107 | 98 | 2 | 2 | 775 | 98 | 4089 | 1 |
| 1 | 749 | -42 | 134 | 40 | 10 | 2 | 2 | 174 | 10 | 2652 | 0 |
| 1 | 749 | -14 | 1446 | 149 | 44 | 2 | 2 | 1596 | 44 | 8768 | 0 |
| 0 | 802 | -42 | 306 | 67 | 59 | 1 | 2 | 373 | 59 | 2460 | 1 |
| 0 | 802 | -14 | 434 | 50 | 65 | 1 | 2 | 483 | 65 | 3577 | 1 |
| 1 | 810 | -42 | 387 | 105 | 6 | 2 | 2 | 492 | 6 | 1625.4 | 0 |
| 1 | 810 | -14 | 418 | 126 | 75 | 2 | 2 | 545 | 75 | 2953.2 | 0 |
| 0 | 825 | -42 | 379 | 108 | 32 | 2 | 2 | 487 | 32 | 3031 | 1 |
| 0 | 825 | -14 | 148 | 25 | 21 | 2 | 2 | 173 | 21 | 3359.4 | 1 |
| 0 | 833 | -42 | 315 | 54 | 23 | 1 | 2 | 369 | 23 | 1431 | 1 |
| 0 | 833 | -14 | 328 | 67 | 35 | 1 | 2 | 395 | 35 | 3013.4 | 1 |
| 1 | 861 | -42 | 280 | 175 | 16 | 2 | 2 | 456 | 16 | 1374.7 | 1 |
| 1 | 861 | -14 | 749 | 84 | 34 | 2 | 2 | 833 | 34 | 5700 | 1 |
| 0 | 903 | -42 | 449 | 73 | 30 | 1 | 2 | 522 | 30 | 5056 | 1 |
| 0 | 903 | -14 | 162 | 21 | 16 | 1 | 2 | 183 | 16 | 6363 | 1 |
| 0 | 905 | -42 | 103 | 17 | 11 | 2 | 2 | 119 | 11 | 3572.8 | 0 |
| 0 | 905 | -14 | 555 | 147 | 93 | 2 | 2 | 701 | 93 | 4991.3 | 0 |
| 1 | 911 | -42 | 139 | 25 | 5 | 1 | 2 | 164 | 5 | 896 | 0 |
| 1 | 911 | -14 | 465 | 128 | 27 | 1 | 2 | 593 | 27 | 2556 | 0 |
| 0 | 913 | -42 | 394 | 65 | 29 | 1 | 2 | 459 | 29 | 3120 | 0 |
| 0 | 913 | -14 | 256 | 33 | 18 | 1 | 2 | 289 | 18 | 1665 | 0 |
| 1 | 926 | -42 |  |  |  | 1 | 2 |  |  |  | 1 |
| 1 | 926 | -14 | 395 | 29 | 47 | 1 | 2 | 424 | 47 | 2639.4 | 1 |
| 1 | 930 | -42 | 675 | 143 | 50 | 2 | 1 | 818 | 50 | 3500 | 1 |
| 1 | 930 | -14 | 949 | 133 | 110 | 2 | 1 | 1082 | 110 | 3828.5 | 1 |
