# Supplementary material for: Counts of bovine monocyte subsets prior to calving are predictive for postpartum occurrence of mastitis and metritis
Source: Vet Res. 2017 Feb 21;48:13. doi: 10.1186/s13567-017-0415-8 (PMC5320682; doi:10.1186/s13567-017-0415-8)
Supplement: Supplementary file 2 — Additional file 2. Cell counts by parity. This file contains a table summarizing descriptive analysis of cell count by parity group (Results). [file 13567_2017_415_MOESM2_ESM.docx]

**Additional file 2 Cell counts by parity.** Peripheral blood monocyte and neutrophil cell counts (cells/μL) grouped by parity and disease status. Median counts with interquartile range (IQR) presented.

| Healthy | | | | | | |
| --- | --- | --- | --- | --- | --- | --- |
| Parity > 2 (*n* = 9) | Neutrophils (cells/μL) | | CD14- monocytes  (cells/μL) | | CD14+ monocytes (cells/μL) | |
| Days prepartum | **Median** | **IQR** | **Median** | **IQR** | **Median** | **IQR** |
| -42 | 3075.500 | 1468 | 31.08834 | 12.08982 | 433.7490 | 133.34564 |
| -14 | 3468.200 | 2501 | 27.67735 | 40.61797 | 341.9845 | 414.47448 |
|  |  |  |  |  |  |  |
| Parity = 2 (*n* = 5) | **Neutrophils (cells/μL)** | | **CD14- monocytes (cells/μL)** | | **CD14+ monocytes (cells/μL)** | |
| Days prepartum | **Median** | **IQR** | **Median** | **IQR** | **Median** | **IQR** |
| -42 | 3818.200 | 2661 | 68.99881 | 59.69023 | 705.1991 | 561.33173 |
| -14 | 4013.600 | 1305 | 66.50144 | 67.29404 | 602.5972 | 745.55171 |
| Postpartum disease | | | | | | |
| Parity > 2 (*n* = 6) | Neutrophils (cells/μL) | | CD14- monocytes  (cells/μL) | | CD14+ monocytes (cells/μL) | |
| Days prepartum | **Median** | **IQR** | **Median** | **IQR** | **Median** | **IQR** |
| -42 | 1625.400 | 1277 | 8.665826 | 3.84636 | 174.1679 | 291.22023 |
| -14 | 5328.000 | 2747 | 34.17388 | 15.10259 | 757.2828 | 240.06350 |
|  |  |  |  |  |  |  |
| Parity = 2 (*n* = 7) | **Neutrophils (cells/μL)** | | **CD14- monocytes (cells/μL)** | | **CD14+ monocytes (cells/μL)** | |
| Days prepartum | **Median** | **IQR** | **Median** | **IQR** | **Median** | **IQR** |
| -42 | 3135.600 | 1057 | 43.90255 | 14.47735 | 677.4227 | 307.80345 |
| -14 | 4212.100 | 1612 | 63.93440 | 59.29398 | 1070.528 | 219.94237 |
